# Development and evaluation of chitosan nanocapsules co-loaded with tea tree oil and thymol: Physicochemical characterization, pH-responsive release, and *in vitro* antibacterial activity against mastitis-associated pathogens

**DOI:** 10.14202/vetworld.2026.1954-1969

**Published:** 2026-05-12

**Authors:** Lysett Corona-Gómez, María de la Luz Zambrano-Zaragoza, Alicia del Real, Samantha Jardon Xicotencatl, Laura Hernández-Andrade, Lizbeth Martínez-Acevedo, Susana Mendoza-Elvira, David Quintanar-Guerrero

**Affiliations:** 1 Graduate Laboratory in Pharmaceutical Technology, FES-Cuautitlán, National Autonomous University of Mexico (UNAM), State of Mexico, Mexico; 2 Laboratory of Food Processing and Emerging Technologies, FES-Cuautitlán, National Autonomous University of Mexico (UNAM), State of Mexico, Mexico; 3 Department of Molecular Materials Engineering, Center for Applied Physics and Advanced Technology, National Autonomous University of Mexico (UNAM), Juriquilla Campus, Santiago de Querétaro, Mexico; 4 Laboratory of Veterinary Morphology and Cellular Biology, Multidisciplinary Research Unit, FES-Cuautitlán, National Autonomous University of Mexico (UNAM), State of Mexico, Mexico; 5 Bacteriology Laboratory, National Center for Disciplinary Research in Animal Health and Food Safety (CENID-SAI), National Institute for Forestry, Agricultural and Livestock Research (INIFAP), Mexico City, Mexico; 6 Galenic Development Laboratory, Department of Biological Systems, Autonomous Metropolitan University (UAM), Xochimilco Unit, Mexico City, Mexico; 7 Laboratory of Microbiology and Virology of Swine Respiratory Diseases, FES-Cuautitlán, National Autonomous University of Mexico (UNAM), State of Mexico, Mexico

**Keywords:** antibacterial activity, bovine mastitis, chitosan nanocapsules, essential oils, ionic gelation, nanotechnology, pH-responsive release, thymol, tea tree oil

## Abstract

**Background and Aim:**

Bovine mastitis remains one of the most economically significant diseases in dairy production and is a major driver of antimicrobial usage. The increasing prevalence of antimicrobial resistance has necessitated the development of alternative therapeutic strategies. Essential oils (EOs) and nanotechnology-based delivery systems have emerged as promising non-antibiotic approaches. This study aimed to develop chitosan nanocapsules (NC) co-loaded with tea tree oil (TTO) and thymol, and to evaluate their physicochemical characteristics, cytocompatibility, pH-responsive release behavior, and *in vitro* antibacterial activity against mastitis-associated pathogens.

**Materials and Methods:**

Chitosan NC containing TTO and thymol were prepared using the ionic gelation method. Physicochemical properties were assessed by dynamic light scattering, scanning electron microscopy, and zeta potential analysis. Cytotoxicity was evaluated using MARC-145 cells. *In vitro* release studies were performed under simulated bovine mammary pH conditions (pH 6.6 and 7.2). Antibacterial activity was tested against *Escherichia coli* and *Staphylococcus aureus* using growth kinetics assays.

**Results:**

The NC exhibited a mean particle size of 383.1 ± 10.2 nm, a polydispersity index of 0.20, and a positive zeta potential of 24.7 ± 1.3 mV, indicating good stability. Encapsulation efficiency reached 77.5 ± 2.3%. Cytotoxicity analysis showed improved cell viability for nanoformulations compared with free oils, particularly for nanoemulsions. Release studies demonstrated a clear pH-dependent behavior, with limited release at pH 6.6 (18.4%) and enhanced release at pH 7.2 (61.7%) over 70 h. Antibacterial assays revealed that chitosan NC achieved the highest inhibitory effect, reducing *S. aureus* and *E. coli* growth by 92% and 72%, respectively. The antibacterial activity against *S. aureus* was comparable to erythromycin under the tested conditions.

**Conclusion:**

Chitosan NC co-loaded with TTO and thymol demonstrated enhanced antibacterial activity, controlled-release, and improved cytocompatibility under *in vitro* conditions. The pH-responsive release profile highlights their potential as a targeted intramammary delivery system for mastitis management. These findings support further *in vivo* investigations to validate their applicability as a non-antibiotic alternative in dairy production systems.

## INTRODUCTION

Bovine mastitis remains one of the costliest diseases affecting dairy cattle [1] and accounts for approximately 70% of antibiotic use on dairy farms [2]. This intensive use of antimicrobials has contributed significantly to the emergence and spread of antibiotic-resistant bacterial strains, posing a serious challenge to animal health, public health, and dairy sustainability [3]. In this context, the development of effective, environmentally friendly, and non-antibiotic alternatives for controlling bacterial infections has become an urgent priority [4].

Among the proposed alternatives, essential oils (EOs) have attracted increasing attention due to their broad antimicrobial and antioxidant properties. These plant-derived compounds are composed of multiple bioactive constituents and are widely used in the formulation of aromas, foods, and pharmaceutical products [5]. However, despite their promising biological activity, the direct application of EOs is limited by their volatility, strong aroma, low water solubility, and susceptibility to degradation under adverse environmental conditions, such as light, oxygen, humidity, heat, pressure, and chemical exposure.

To overcome these limitations, nanotechnology has emerged as a powerful multidisciplinary approach to improve the delivery and performance of bioactive compounds. Nanoencapsulation systems enable targeted delivery and controlled or sustained release, enhance physicochemical stability, mask undesirable aromas, and protect active compounds from degradation and oxidation. In this regard, non-toxic and highly biocompatible macromolecules have been explored as nanocarriers for active delivery. Lipid- and polymer-based nanosystems, including nanoemulsions (NE), liposomes, and solid lipid nanocapsules (NC), have been successfully employed for the encapsulation of plant-derived EOs [6].

Chitosan, a natural polysaccharide obtained from crustacean exoskeletons, has gained particular interest due to its antimicrobial activity, biocompatibility, biodegradability, and ability to form stable NC, making it suitable for food, pharmaceutical, and biomedical applications [7]. Tea tree oil (TTO) and thymol are natural compounds with well-documented antimicrobial and antioxidant properties [8]. Previous studies have reported that encapsulating TTO and thymol within chitosan-based NC improves their stability and antimicrobial efficacy compared with their free forms [9–12]. These nanosystems have demonstrated inhibitory activity against pathogenic bacteria such as *Salmonella* spp., *Escherichia coli*, and *Staphylococcus aureus*, highlighting their potential as alternatives to conventional antimicrobial agents [13, 14]. Additionally, chitosan-EO NC have been explored for food preservation, where they have been shown to extend shelf life by inhibiting microbial growth in products such as meat and fruit [6].

Ionic gelation is a widely used method for preparing chitosan NC due to its simplicity, mild processing conditions, and compatibility with bioactive compounds. This technique involves electrostatic interactions between chitosan and a polyanion, such as sodium tripolyphosphate (TPP), resulting in the formation of uniform, controllable NC without requiring extreme pH, temperature, or pressure, thereby minimizing degradation of sensitive compounds [15, 16]. While several studies have focused on encapsulating individual EOs, limited information is available on the combined encapsulation of EOs in combination with phenolic metabolites such as thymol or carvacrol.

Despite the growing interest in EO-based nanocarriers, most previous studies have focused on the encapsulation of single EOs and their applications in food preservation, human pathogens, or general antimicrobial systems. In contrast, limited information is available regarding the combined nanoencapsulation of complementary EO compounds for veterinary applications, particularly for the control of bovine mastitis. TTO is a complex mixture of terpene compounds with broad antimicrobial activity, whereas thymol is a phenolic monoterpene known for its strong membrane-disrupting properties. Previous work from our research group demonstrated the antimicrobial activity of free TTO, thymol, and carvacrol against bacteria isolated from bovine clinical mastitis [10]. However, to the best of our knowledge, the co-encapsulation of TTO and thymol within chitosan NC and their evaluation as a potential intramammary delivery system for mastitis management have not been reported previously.

Furthermore, mastitis is associated with physiological changes in mammary gland pH due to inflammation and increased permeability of the blood–milk barrier, which may influence the release of therapeutic compounds. The development of nanocarriers capable of pH-responsive release under mastitis-associated conditions could therefore represent an innovative strategy to improve the local availability of antimicrobial agents during infection while maintaining controlled-release under normal physiological conditions.

Although EOs and nanotechnology-based delivery systems have demonstrated considerable potential as alternative antimicrobial strategies, current research is largely limited to encapsulating single EOs and evaluating them in food preservation or human-related microbial systems. There is a lack of studies focusing on the combined nanoencapsulation of complementary EO compounds with synergistic antimicrobial mechanisms, particularly in veterinary applications such as bovine mastitis. In addition, limited attention has been given to the development of pH-responsive nanocarriers that can adapt to physiological changes associated with mastitis, such as increased mammary gland pH during infection. Moreover, the cytocompatibility and controlled-release behavior of such co-encapsulated systems under *in vitro* conditions relevant to mastitis remain insufficiently explored.

Therefore, the aim of this study was to develop and characterize chitosan-based NC loaded with TTO and thymol, and to evaluate their physicochemical, morphological, and thermal properties, as well as their antimicrobial activity and *in vitro* release behavior. We hypothesized that encapsulating TTO and thymol within chitosan NC would enhance antimicrobial efficacy and biocompatibility while enabling pH-dependent release under mastitis-simulated conditions, compared with free EOs and non-encapsulated chitosan.

## MATERIALS AND METHODS

### Ethical approval

Ethical approval was not required for this study because no live animals were directly used. All experimental procedures were conducted using established cell lines and reference bacterial strains under controlled *in vitro* laboratory conditions, in accordance with institutional biosafety and laboratory practice guidelines.

### Study period and location

The study was conducted from August 2020 to December 2024 at the Graduate Laboratory in Pharmaceutical Technology and associated research laboratories of the National Autonomous University of Mexico (UNAM), State of Mexico, Mexico. All experimental procedures, including formulation development, physicochemical characterization, microbiological assays, and *in vitro* evaluations, were performed under controlled laboratory conditions.

### Study design

This study employed a laboratory-based experimental design to develop and evaluate chitosan-based NC co-loaded with TTO and thymol. The study included formulation development using the ionic gelation method, physicochemical characterization, analytical method validation, determination of encapsulation efficiency, cytocompatibility assessment, *in vitro* release studies under simulated bovine mammary pH conditions, and antibacterial activity evaluation against mastitis-associated pathogens.

### Materials

TTO (*Melaleuca alternifolia*) (lot 2023313; manufacturer details not provided) and thymol (≥98.5%; Sigma-Aldrich, St. Louis, MO, USA) were used as active compounds. Both products were of pharmaceutical grade and were supplied with technical data sheets and certificates of analysis provided by the manufacturer. The EOs were used as received, without further purification.

Chitosan (low molecular weight with molecular weight of 50–90 kDa, degree of deacetylation <70%; Sigma-Aldrich, St. Louis, MO, USA), sodium tripolyphosphate (TPP; Sigma-Aldrich), Tween 80 (Sigma-Aldrich), and glacial acetic acid (Sigma-Aldrich) were of analytical grade and used as received. All aqueous solutions were prepared using distilled water.

### Microbial strains and culture media

Reference strains of *E. coli* (ATCC® 8739™) and *S. aureus* (ATCC® BAA-976™) were used for antimicrobial assays. Both strains were cultured in brain–heart infusion (BHI) medium and incubated under aerobic conditions at 37°C prior to experimental use.

### Preparation of chitosan NC loaded with EOs

Chitosan NCs were prepared using the ionic gelation method, following the protocol described by Hoang *et al*. [7], with slight modifications to adapt the system for encapsulating TTO and thymol.

Briefly, a chitosan solution was prepared by dissolving chitosan at 0.3% (w/v) in 1% (v/v) acetic acid under magnetic stirring until complete dissolution. Tween 80 (Merck, Darmstadt, Germany) was then added as a stabilizing agent at a final concentration of 1% (v/v). Separately, a Sodium tripolyphosphate (TPP, Merck, Darmstadt, Germany) solution was prepared at 0.3% (w/v) in distilled water. TTO and thymol (94% and 6%, respectively) were incorporated into the chitosan solution by dropwise addition over 15 min under continuous magnetic stirring.

The pH of the mixture was adjusted to 4.5 using sodium hydroxide solution. Subsequently, the TPP solution was added dropwise at a controlled flow rate of 1 mL/min, promoting nanoparticle formation through electrostatic interactions between chitosan and TPP. A chitosan:TPP mass ratio of 3:1 was used. The resulting suspension was stirred for 30 min to ensure the complete formation of NC. Purification was performed by centrifugation at 6,000 rpm (4830 × *g*) for 20 min. This ionic gelation process was selected for its simplicity, mild processing conditions, and compatibility with sensitive bioactive compounds, enabling nanoparticle formation without extreme pH, temperature, or pressure. The methodology was adapted from Hoang *et al*. [7] to optimize encapsulation of EOs and phenolic metabolites.

### Characterization of chitosan NC

**Particle size (PS), polydispersity index (PDI), and zeta potential (ZP):** PS and PDI of the chitosan NC were determined by dynamic light scattering using a Zetasizer (ZEN3600, Malvern Instruments Ltd., Malvern, UK). ZP was measured using the same equipment based on the electrophoretic mobility of particles in suspension, employing polystyrene dispersions as a reference (ζ = −55 mV).

Prior to analysis, nanocapsule suspensions were diluted in distilled water to an appropriate ratio to avoid multiple scattering. Measurements were performed at 25°C, with a detection angle of 90°. Each batch was analyzed in six independent measurements, and results were expressed as mean ± standard deviation.

### Morphological characterization

The morphology of the chitosan NC was examined by scanning electron microscopy (SEM). A drop of the NC suspension was placed onto a sample holder and dried in a refrigerated desiccator. The samples were subsequently coated with a thin gold layer (approximately 2 nm) using a fine coat ion sputter deposition unit (JFC-1100, JEOL Ltd., Tokyo, Japan).

SEM observations were performed using a JSM-5600 LV scanning electron microscope (JEOL Ltd.) operated at 28 kV, with a resolution of 5 nm and a chamber pressure of 12-20 Pa.

### Validation of the analytical method

**Absorption spectra and selection of analytical conditions:** The wavelengths of maximum absorption were determined for TTO and thymol by scanning standard solutions prepared in methanol. Absorption spectra were recorded over a wavelength range of 200–500 nm to identify the optimal detection wavelength for chromatographic analysis. To optimize chromatographic separation, different proportions of acetonitrile and water were evaluated as mobile phase. The selected mobile phase provided adequate peak resolution from potential interferences, optimal peak symmetry, and minimal retention time for the analytes. The injection volume was set at 30 μL, and the flow rate was fixed at 1.0 mL/min, based on preliminary optimization tests.

**Chromatographic conditions and calibration curves:** Chromatographic analysis was performed using a ProStar 410 HPLC system equipped with a Varian® 100 RP-18 (250 × 4.6 mm, 5 μm; Varian Inc., Palo Alto, CA, USA) column under isocratic conditions. The mobile phase consisted of acetonitrile and water (70:30, v/v). Detection was carried out at a wavelength of 270 nm. The flow rate was maintained at 1 mL/min, and the injection volume was set at 30 μL. All analyses were performed under standardized conditions to ensure reproducibility. Calibration curves were constructed using six concentration levels ranging from 0.1 to 100 μg/mL for both TTO and thymol. All calibration curves were prepared and analyzed on the same day under identical chromatographic conditions. The analytical response was obtained by plotting the peak area against the corresponding analyte concentration. The slope and intercept were calculated for each calibration curve to evaluate linearity. Terpinen-4-ol, the major bioactive component of TTO, was used as the reference standard for TTO quantification. All analyses were performed in triplicate.

**Validation parameters:** Method validation was carried out for linearity, based on calibration curves, and for repeatability, assessed by triplicate injections at each concentration level. The method demonstrated acceptable linearity within the evaluated concentration range. Due to the scope of this study, other validation parameters such as limit of detection, limit of quantification, accuracy, and robustness were not evaluated.

**Linearity:** Linearity was evaluated over the concentration range of 0.1–100 μg/mL for both TTO and thymol. Calibration curves were constructed by plotting peak area versus analyte concentration. Linear regression analysis showed excellent linearity, with correlation coefficients of ≥0.99 for both compounds. The slopes and intercepts demonstrated a consistent proportional response across the tested range.

**Limit of detection and limit of quantification:** The limit of detection and limit of quantification were theoretically estimated from the calibration curves in accordance with ICH Q2(R1) guidelines, using the standard deviation of the response (σ) and the slope of the calibration curve (S), according to the following equations:

LOD = (3.3 × σ) / S

LOQ = (10 × σ) / S

where σ corresponds to the standard deviation of the y-intercepts of the regression lines and S represents the slope of the calibration curve. These values provide an estimate of the method’s sensitivity suitable for comparative quantification and determination of encapsulation efficiency.

**Repeatability (precision):** Repeatability was evaluated using triplicate measurements at each concentration. The method showed acceptable precision, with coefficient of variation values ranging from 0.004 to 0.10 for TTO and from 0.008 to 0.09 for thymol, confirming good intra-day precision.

**Scope and limitations of the validation:** This validation approach was designed to support comparative quantification of free and encapsulated compounds rather than full pharmaceutical validation. Therefore, parameters such as accuracy, robustness, selectivity, and inter-day precision were not evaluated, as they were beyond the scope of the present study.

### Determination of encapsulation efficiency

The encapsulation efficiency (EE) of TTO and thymol was determined using an indirect method based on quantifying non-encapsulated compounds in the supernatant, as described in previous studies [17, 18]. Following centrifugation at 6,000 rpm (4830 × *g*) for 60 min, a 2 mL aliquot of the supernatant was analyzed by high-performance liquid chromatography. The amount of free oil was quantified using calibration curves, and the encapsulated fraction was calculated by difference.

EE was calculated as follows:

EE (%) = (Total oil added − Free oil detected) / Total oil added × 100

All determinations were performed in triplicate, and results were expressed as mean ± standard deviation.

### Morphological analysis by immunofluorescence

Immunofluorescence analysis was performed to assess potential alterations in cytoskeletal organization following exposure to the nanoformulations. This approach complements the 3-(4,5-dimethylthiazol-2-yl)-2,5-diphenyltetrazolium bromide (MTT) assay by providing qualitative information on cellular morphology and cytoskeletal integrity. To evaluate morphology-associated cellular changes, 1 × 10^5^ cells were seeded onto sterilized 12 mm glass coverslips and exposed for 24 h to 0.3% chitosan NC loaded with TTO and thymol (QS/TTO/Thymol; NC), 0.3% NE, or 0.3% free EOs, under standard culture conditions.

After treatment, cytoskeletal organization was assessed by direct immunofluorescence staining. Cells were fixed with 10% (v/v) aqueous formalin for 20 min, followed by permeabilization with 0.5% (v/v) Triton X-100. The samples were then incubated with rhodamine isothiocyanate–conjugated phalloidin for 20 min in a light-protected chamber to visualize filamentous actin. Nuclei were counterstained using 4′,6-diamidino-2-phenylindole. Between each step, samples were washed three times with phosphate-buffered saline. Coverslips were mounted onto glass slides and analyzed by fluorescence microscopy to evaluate alterations in cell morphology and cytoskeletal integrity associated with treatment exposure.

### *In vitro* cell line evaluation and cytotoxicity assay

MARC-145 cells were cultured in Dulbecco’s Modified Eagle Medium supplemented with 10% fetal bovine serum, penicillin (5000 IU/mL), and streptomycin (5 μg/mL). Cells were maintained in a humidified incubator at 37°C under an atmosphere of 95% air and 5% carbon dioxide. Prior to cell exposure, all formulations were sterilized by ultraviolet irradiation for 4 h at room temperature.

Cytotoxicity was evaluated using the MTT assay. Briefly, 1 × 10^4^ cells per well were seeded into 96-well plates and allowed to adhere for 24 h. Cells were then exposed to the different formulations for 12, 24, and 48 h. Following the exposure periods, the culture medium was removed, and cells were washed with sterile phosphate-buffered saline. Subsequently, MTT solution (0.5 mg/mL) was added to each well, and the plate was incubated for 3 h at 37°C. After incubation, the supernatant was carefully removed, and the formazan crystals were solubilized with 100 μL of dimethyl sulfoxide per well. Absorbance was measured at 570 nm using an enzyme-linked immunosorbent assay microplate reader. All assays were performed in triplicate.

For morphological assessment, treated cells were maintained in the dark and observed using a Zeiss Axio Scope 40® fluorescence microscope. Ten random fields were captured per sample using 20× and 40× objectives, and images were independently analyzed using ImageJ software (National Institutes of Health, Bethesda, MD, USA).

### *In vitro* release study using Franz diffusion cells under bovine mammary pH conditions

*In vitro* release studies were performed using Franz diffusion cells to simulate the pH conditions of the bovine mammary gland in healthy and mastitic states. The diffusion cells were maintained at 39.0 ± 0.5°C. A volume of 10 mL of 0.3% chitosan nanocapsule suspension (QS/TTO/Thymol; NC) or 0.3% NE was placed in the donor compartment.

The receiver compartment contained buffer solution supplemented with 0.5% Tween 80 to maintain sink conditions for poorly soluble EOs. The receiver compartment was filled with 10 mL of buffer solution at pH 6.6 or pH 7.2. A semi-permeable cellulose acetate membrane was used to separate the donor and receptor compartments.

At predetermined time intervals (15, 30, 60, 120, 180, 240, 300, 360, 420, 480, and 960 min), 1 mL aliquots were withdrawn and replaced with fresh buffer. The concentration of active compounds released was quantified by high-performance liquid chromatography. Release profiles were expressed as cumulative release over time. Release kinetics were evaluated using zero-order, first-order, Higuchi, and Korsmeyer–Peppas models.

### *In vitro* antibacterial activity

The *in vitro* antibacterial efficacy of the different formulations was evaluated using a bacterial growth kinetics assay, based on turbidity measurements over time. Reference strains of *E. coli* (ATCC® 8739™) and *S. aureus* (ATCC® BAA-976™) were used as representative Gram-negative and Gram-positive mastitis-associated pathogens, respectively. Briefly, 25 mL of brain–heart infusion (BHI, BD, Franklin Lakes, NJ, USA) broth was dispensed into sterile Erlenmeyer flasks. Each flask was inoculated with 600 μL of bacterial suspension adjusted to 0.5 McFarland standard, corresponding to an optical density of approximately 0.1 at 600 nm (OD600). Subsequently, 600 μL of the different treatments were added as follows: 0.3 % chitosan NC loaded with tea tree oil and thymol (QS/TTO/Thymol; NC), 0.3 % NE, chitosan NC at 0.3 % and 0.6 %, and free essential oils at 0.3 % and 0.6 %. Erythromycin (Merck, Darmstadt, Germany)was included as an antibiotic reference control. Control groups consisted of a positive control (BHI medium with bacterial inoculum and no treatment), a negative control (formulations without bacterial inoculum), and a sterility control (BHI medium only). All flasks were incubated at 37°C under constant agitation. Bacterial growth was monitored by measuring turbidity at 600 nm at 0, 2, 4, 24, and 27 h using a spectrophotometer. All experiments were conducted in triplicate, and growth curves were generated for each treatment and bacterial strain.

### Statistical analysis

Statistical analyses were performed using IBM SPSS Statistics software (IBM Corp., Armonk, NY, USA). Data normality was assessed using the Shapiro–Wilk test, and homogeneity of variances was evaluated using Levene’s test. For normally distributed data, two-way analysis of variance (ANOVA) followed by Tukey’s post hoc test was used. For non-normally distributed data, the Kruskal–Wallis test was followed by Dunn’s multiple-comparison test. Results are expressed as mean ± standard deviation, and statistical significance was established at p < 0.05.

## RESULTS

### PS, PDI, and ZP

Table 1 summarizes PS, PDI, and ZP of the different formulations, including mean values and standard deviations obtained from six independent measurements. ANOVA showed no statistically significant differences among batches (p > 0.05), indicating good reproducibility of the nanoparticle preparation process. All chitosan-based formulations exhibited particle sizes in the submicron range, with PDI values ≤0.3, indicating a relatively narrow size distribution. ZP values were positive for all chitosan-containing systems, reflecting the cationic nature of chitosan and suggesting electrostatic stability of the dispersions. In contrast, the TTO–thymol NE exhibited a smaller PS and a lower ZP than the chitosan-based NC.

**Table 1:** PS, PDI, and ZP of the different formulations.

Formulation	PS (nm)	PDI	ZP (mV)
Chitosan	347.3 ± 16.3ᵃ	0.20 ± 0.06ᵃ	30.3 ± 1.1ᵃ
TTO–thymol NE	20.8 ± 2.8ᵇ	0.30 ± 0.10ᵇ	9.0 ± 1.1ᵇ
Chitosan–TTO	398.3 ± 0.3ᶜ	0.30 ± 0.06ᵇ	30.9 ± 0.9ᵃ
Chitosan–thymol	287.1 ± 57.1ᵃ	0.20 ± 0.01ᵃ	28.7 ± 0.8ᵃ
Chitosan–TTO–thymol	383.1 ± 10.2ᶜ	0.20 ± 0.01ᵃ	24.7 ± 1.3ᶜ

PS = Particle size, PDI = Polydispersity index, ZP = Zeta potential. Data are presented as mean ± standard deviation (n = 6). Different superscript letters within the same column indicate significant differences (p < 0.05).

### Morphological characterization of NC

**Figure 1:**
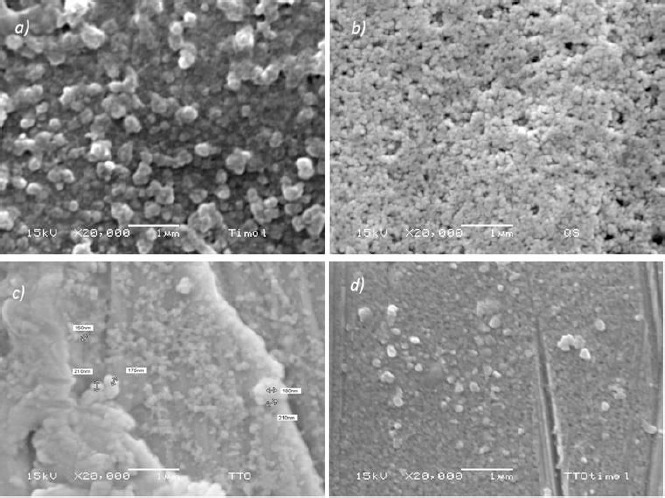
Morphological characterization of polymeric NC by SEM. Representative micrographs show chitosan-based NC: (a) chitosan NC, (b) chitosan + thymol, (c) chitosan + TTO, and (d) chitosan + thymol + TTO. The particles exhibited predominantly spherical morphology with submicron size (<1 μm) and a tendency to aggregate, particularly in formulations without encapsulated compounds. Differences in surface morphology were observed depending on the active compounds incorporated. Images were obtained at 15 kV and 20,000× magnification. Scale bar: 1 μm.

The micrographs of the chitosan polymeric NC loaded with TTO and thymol revealed apparently spherical structures, with some distortion and the presence of agglomerates (Figure 1). The observed NC exhibited a submicron size, consistent with PS analysis. Alongside regions showing nanoparticle agglomeration, individual NC and small clusters were also detected, particularly in the early stages of nanoparticle formation. In contrast, chitosan NC without EOs displayed an apparently spherical but more flattened morphology, with smaller particles embedded within larger agglomerates. The incorporation of EOs appeared to enhance nanoparticle sphericity and colloidal stability, as evidenced by reduced aggregation and improved visualization of individual particles. These observations suggest that the presence of TTO and thymol may influence nanoparticle organization and surface characteristics.

### *In vitro* evaluation of cell viability

Preliminary dose–response screening indicated that 50 μL per well was the highest dose that did not induce a cytotoxic effect, defined as cell viability ≥75%. This dose was therefore selected for subsequent cytotoxicity evaluation at 12, 24, and 48 h. At 12 h of exposure, no significant differences in cell viability were observed among the treated groups compared with the control. At longer exposure times (24 and 48 h), differences among formulations became evident. After 24 h, all tested systems maintained cell viability above the cytotoxicity threshold. However, after 48 h, NE was the only formulation that preserved cell viability above 75% (83%), whereas cultures treated with NC and free oils showed a marked reduction in viability (64% and 56%, respectively). At 24 h, no significant differences were observed between control and oil-treated cells (p = 0.094), whereas NE and NC showed a slight but significant reduction in cell viability (p = 0.041). At 48 h, significant differences among treatments were observed (p < 0.001), with NE maintaining higher viability compared with NC and free oils (p = 0.006 and p = 0.003, respectively) (Table 2).

**Table 2:** Effect of treatments on cell viability (%) at different time points.

Time (h)	Control	NE	NC	Oils
24	100ᵃ	87.0 ± 0.17ᵇ	82.0 ± 0.11ᵇ	100.0 ± 0.11ᵃ
48	100ᵃ	83.0 ± 0.30ᵇ	64.0 ± 0.20ᶜ	56.0 ± 0.30ᶜ

NE = Nanoemulsion, NC = Nanocapsules. Data are presented as mean ± standard deviation (n = 3). Different superscript letters within the same row indicate statistically significant differences among treatments (p < 0.05).

### Morphological evaluation of actin cytoskeleton

**Figure 2:**
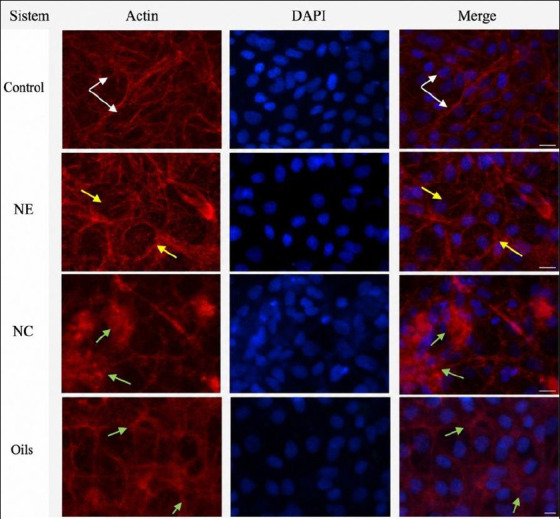
Actin cytoskeleton organization after exposure to different formulations.

Morphological assessment was performed by analyzing the actin cytoskeleton organization. Cells exposed to NE for 48 h exhibited a morphology comparable to that of control cells, characterized by well-defined and homogeneous actin filaments at the perinuclear and cortical regions (Figure 2, white arrows). Stress fibers extended radially across the cytoplasm toward the cell cortex (Figure 2, yellow arrows), with centrally positioned nuclei and clearly defined cell contours. In contrast, cells treated with NC and free EOs showed alterations in cytoskeletal organization, including partial depolymerization of actin filaments, particularly at the level of stress fibers (Figure 2, green arrows).

### Analytical method validation

The analytical method showed good linearity for both TTO and thymol within the concentration range of 1–100 μg/mL. Linear regression analysis of the calibration curves demonstrated correlation coefficients ≥0.99 for both analytes, indicating a strong proportional relationship between concentration and chromatographic response. Repeatability was evaluated by triplicate analysis at each concentration level. The method showed acceptable intra-day precision, with coefficient of variation values ranging from 0.004 to 0.10 for TTO and from 0.008 to 0.09 for thymol, confirming the consistency of the analytical response under the evaluated conditions. The limit of detection and limit of quantification were estimated from the calibration curve parameters according to ICH Q2(R1) guidelines, using the standard deviation of the response and the slope of the calibration curve. The calculated limit of detection and limit of quantification values were 0.52 and 1.58 μg/mL for thymol and 0.61 and 1.85 μg/mL for TTO, respectively, confirming adequate sensitivity of the method within the studied concentration range. The results confirm that the method provides adequate sensitivity for quantifying the active compounds within the concentration range studied. Terpinen-4-ol was used as a reference compound to identify the characteristic peak of TTO based on retention time comparison. Quantification of TTO was performed based on the overall chromatographic response of the oil. The validation parameters obtained for both analytes are summarized in Table 3.

**Table 3:** Summary of analytical method validation parameters for tea tree oil (TTO) and thymol determined by high-performance liquid chromatography (HPLC).

Parameter	TTO	Thymol
Linearity range (μg/mL)	1–100	1–100
Correlation coefficient	≥0.99	≥0.99
Coefficient of variation (%)	0.004–0.10	0.008–0.09
Regression model	y = mx + b	y = mx + b
Analytical technique	HPLC	HPLC

Calibration curves were constructed from six concentration levels analyzed in triplicate. Precision was expressed as intra-day repeatability under the same chromatographic conditions.

### EE

EE of the chitosan NC was determined by quantifying the non-encapsulated active compounds present in the supernatant after nanoparticle separation. From each nanoparticle colloidal dispersion (60 mL total volume), an aliquot of 2 mL of supernatant was collected and analyzed by HPLC. Based on the indirect quantification of the free active compounds and the initial amount added, the calculated EE was 77.5 ± 2.3% (Table 4), indicating a high incorporation of TTO and thymol within the chitosan NC.

**Table 4:** Concentration of active compounds and EE determined by the indirect method.

Parameter	Value (mean ± SD)
Initial concentration (mg/mL)	550.0
Supernatant concentration (mg/mL)	126.29 ± 3.7
EE (%)	77.5 ± 2.3

Values are presented as mean ± standard deviation (n = 6).

### Franz cell release testing under bovine mammary gland pH conditions

**Figure 3:**
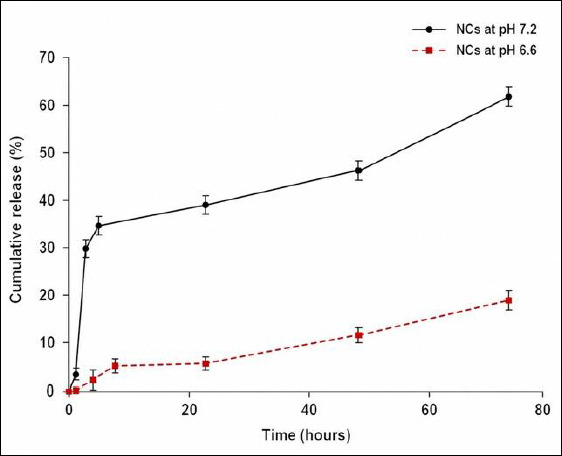
Release profile of NC under different pH conditions (7.2 and 6.6). Data are presented as mean ± standard deviation. Significant differences were observed between pH conditions (p < 0.001), indicating a pH-dependent release behavior.

**Figure 4:**
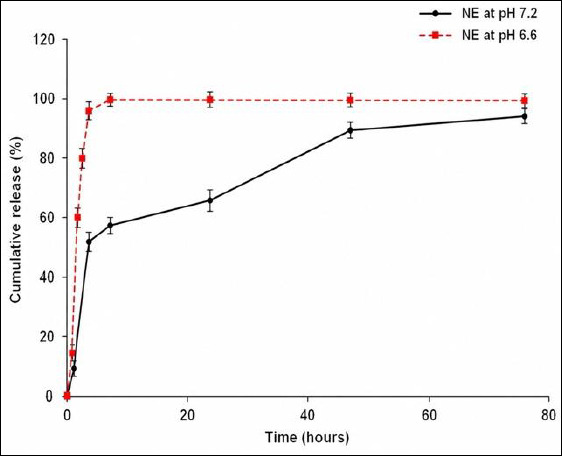
Release profile of NE under different pH conditions (7.2 and 6.6). Data are presented as mean ± standard deviation. A rapid release was observed, particularly at pH 6.6, where nearly complete release occurred within the first hours. Significant differences were observed between pH conditions (p < 0.001), indicating a faster release at acidic pH.

The *in vitro* release kinetics of active compounds from NC and NE under simulated bovine mammary gland pH conditions (pH 6.6 and pH 7.2) over a period of 70 h are shown in Figures 3 and 4, respectively. As shown in Figure 4, NE exhibited rapid diffusion of the active compounds through the membrane at pH 6.6, reaching complete release within the first 5 h of sampling. Under pH 7.2 conditions, NE showed a sustained-release profile, achieving 94.7% cumulative release at 70 h. In contrast, NC exhibited slower, more controlled-release behavior (Figure 3). At pH 6.6, only 18.4% of the encapsulated active compounds were released after 70 h. However, when exposed to pH 7.2, NC released a significantly higher fraction of active compounds, reaching a cumulative release of 61.7% by the end of the assay. Overall, the results demonstrate a clear pH-dependent release behavior, with NC showing enhanced release under conditions simulating intramammary infection compared with physiological mammary gland pH.

### Release kinetics modeling

Table 5 summarizes the mathematical modeling of the release profiles obtained for NC and NE under different pH conditions. Based on the coefficients of determination, the Higuchi model provided the best fit for the release data of NC at both pH 6.6 and pH 7.2, indicating that the release process was predominantly diffusion driven. According to the Korsmeyer–Peppas model, NC exhibited anomalous transport behavior, with release exponent values between 0.6 and 0.83, suggesting the contribution of multiple release mechanisms. In contrast, NE displayed Fickian diffusion behavior, with release exponent values below 0.5 at both pH conditions, consistent with concentration-independent diffusion-controlled release. Overall, these results indicate distinct release mechanisms between NC and NE, depending on formulation type and environmental pH. The first-order model was not included in the final analysis due to its lower fitting performance compared to the zero-order and Higuchi models, indicating that it did not adequately describe the release behavior of the systems.

**Table 5:** Release mechanisms and kinetic models describing NC and NE systems under different pH conditions.

System	k₀	R² (zero-order)	kH	R² (Higuchi)	n	kKP	Release mechanism
NC, pH 7.2	2.15	0.6561	5.84	0.9144	0.83	0.42	Anomalous
NC, pH 6.6	1.18	0.9631	3.92	0.9487	0.60	0.36	Anomalous
NE, pH 7.2	4.72	0.6752	7.18	0.8375	0.20	0.61	Fickian diffusion
NE, pH 6.6	6.53	0.3071	9.05	0.4652	0.10	0.74	Fickian diffusion

NC = Nanocapsules, NE = Nanoemulsion. Release kinetics were analyzed using zero-order, first-order, Higuchi, and Korsmeyer–Peppas models. R² indicates the goodness of fit. The release exponent (n) was used to classify the release mechanism as Fickian diffusion (n ≤0.45) or anomalous transport (0.45 < n < 0.89). Assays were conducted at pH 7.2 and 6.6 to simulate physiological and mastitis-like conditions.

### *In vitro* antibacterial activity against *S. aureus* and *E. coli*

**Figure 5:**
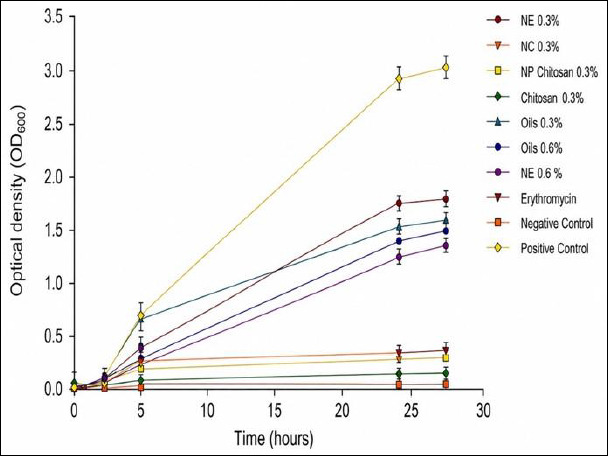
Antibacterial activity of different formulations against *Staphylococcus aureus*, expressed as optical density over time. Treatments included NE, NC, chitosan, and EOs at 0.3% and 0.6%, compared with erythromycin and control groups. Data are presented as mean ± standard deviation. Statistical significance was determined relative to the untreated control at 27 h as follows: *p < 0.05, **p < 0.01, ***p < 0.001, ns = Not significant. NC (0.3%) and erythromycin showed the highest antibacterial activity (***p < 0.001), with no significant differences between them (ns). NE (0.6%) (**p < 0.01) and NE (0.3%) (*p < 0.05) showed moderate activity, while EOs exhibited a concentration-dependent effect. NE = Nanoemulsion, NC = Nanocapsules, EOs = Essential oils.

**Figure 6:**
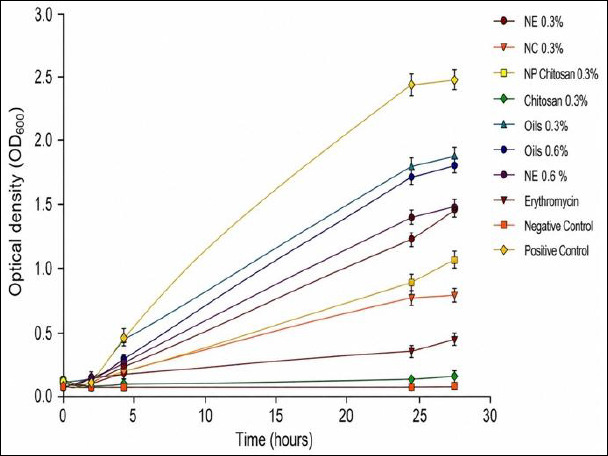
Antibacterial activity of different formulations against Escherichia coli, expressed as optical density over time. Treatments included NE, NC, chitosan, and EOs at 0.3% and 0.6%, compared with erythromycin and control groups. Data are presented as mean ± standard deviation. Statistical significance was determined relative to the untreated control at 27 h as follows: *p < 0.05, **p < 0.01, ***p < 0.001, ns = Not significant. NC (0.3%) exhibited the highest antibacterial activity (***p < 0.001), followed by chitosan (0.3%) and NE (0.6%) (**p < 0.01). NE (0.3%) and EOs showed moderate activity (*p < 0.05), while erythromycin also demonstrated significant inhibition (**p < 0.01). A significant difference was observed between NC and erythromycin (**p < 0.01). NE = Nanoemulsion, NC = Nanocapsules, EOs = Essential oils.

The antibacterial activity of the different formulations against *S. aureus* is shown in Figure 5, with the positive control set at 100% bacterial growth. Treatment with free EOs at concentrations of 0.3% and 0.6% resulted in a 43% reduction in bacterial growth. When the oils were formulated as NE, a 44% reduction was observed at 0.3% NE, whereas 0.6% NE exhibited a greater inhibitory effect, achieving a 54% reduction in bacterial growth. A markedly higher antibacterial effect was observed for chitosan-based systems. The application of 0.3% chitosan nanospheres resulted in a 92% reduction in *S. aureus* growth, which was comparable to the reduction achieved by NC at the same concentration after 27 h of incubation. The bacterial growth kinetics of *E. coli* are shown in Figure 6. Treatment with free EOs at 0.3% and 0.6% resulted in a 28% reduction in bacterial load. In contrast, the application of NE at 0.3% and 0.6% produced a higher inhibitory effect, corresponding to a 40% reduction in bacterial growth. After 27 h, treatment with 0.3% chitosan nanospheres reduced *E. coli* growth by 61%, whereas NC achieved the highest inhibitory effect, with a 72% reduction in bacterial load. Significant differences in antibacterial activity were observed among treatments (p < 0.001). NC showed higher antibacterial activity compared with NE and free oils (p = 0.012 and p = 0.008, respectively). No significant differences were observed between NC and erythromycin against *S. aureus* (p = 0.087), indicating comparable antibacterial performance. For *E. coli*, NC exhibited significantly higher antibacterial activity than NE and free oils (p = 0.018 and p = 0.011, respectively), whereas erythromycin remained significantly more effective (p = 0.004).

## DISCUSSION

### EOs stabilization and NC-based delivery systems

One of the main challenges associated with EOs is preventing the volatilization and degradation of their bioactive components. Among the most effective strategies to overcome these limitations is encapsulation within biopolymeric delivery systems, such as polymeric NCs or NCs [17]. In this context, NC has gained considerable attention due to the biocompatibility, biodegradability, low toxicity, and intrinsic antimicrobial activity of chitosan, as well as its low production cost and suitability for large-scale applications [17].

### PS, PDI, ZP, and EE

The physicochemical characteristics of PS, PDI, ZP, and EE are consistent with previous reports on NCs prepared by the ionic gelation method using TPP as a crosslinking agent [18, 19]. The chitosan/TPP ratio is a critical factor governing NC formation and stability. Stable chitosan-based nanostructures that do not precipitate are commonly obtained at a chitosan/TPP ratio close to 3:1 [20], as TPP contains phosphate groups capable of interacting electrostatically with protonated amino groups of chitosan under acidic conditions.

It has been reported that chitosan molecules can selectively bind to TPP, forming a range of intra- and intermolecular complexes. Consequently, NC formation occurs only under specific conditions of polymer concentration, pH, and chitosan/TPP ratio [21]. At low chitosan concentrations, TPP ions tend to promote coacervation through intermolecular interactions, whereas at polymer concentrations above 0.5 mg/mL and chitosan/TPP ratios greater than 2, nanoparticle or NC formation is favored. Under these conditions, PS increases with increasing chitosan concentration and chitosan/TPP ratio, due to enhanced chain folding and intramolecular crosslinking mediated by tripolyphosphate ions [21].

Importantly, all NC formulations in the present study exhibited PDI values ≤0.3, indicating a narrow size distribution and good colloidal homogeneity. Low PDI values are generally associated with improved physical stability, reduced aggregation, and reproducible performance of nanocarrier systems, which are essential characteristics for controlled-release applications. The positive ZP values observed for NC further support their colloidal stability, as electrostatic repulsion between positively charged particles can prevent aggregation. ZP values greater than ±30 mV are commonly considered indicative of stable colloidal dispersions, and the values obtained for the chitosan-based formulations containing EOs suggest adequate electrostatic stabilization of the systems.

Due to their small molecular size, monoterpenes, such as those present in TTO and thymol, can be efficiently encapsulated within NCs [22]. Previous studies have reported an increase in nanoparticle diameter following EO encapsulation, which is consistent with the results observed in the present work [22]. The EE achieved herein is comparable to values reported for other EO-loaded chitosan systems, including celandine EO [23], thyme EO [24], clove EO [25], and basil EO, with EE ranging from approximately 50% to 75%, depending on formulation parameters.

### Morphological characterization of NC

As shown in Figure 1a, chitosan nanostructures without EOs exhibited a more compact arrangement and an irregular morphology compared with formulations containing oils (Figures 1b–d). In the presence of TTO and thymol, the chitosan-based systems displayed apparently spherical structures, with increased separation between individual NC, facilitating clearer visualization of their morphology. The unloaded chitosan nanostructures appeared more aggregated, whereas EO-loaded NC showed a more defined morphology and reduced clustering.

The aggregation observed in the scanning electron microscopy (SEM) images is consistent with polydispersity index (PDI) values obtained by dynamic light scattering (DLS), indicating a certain degree of heterogeneity in the nanoparticle population. However, it is important to consider that SEM analysis requires sample drying and vacuum conditions, which can promote particle aggregation and may not accurately represent the native colloidal state of the nanoparticles in suspension. In contrast, DLS measurements are performed in aqueous media and provide information on the hydrodynamic diameter and dispersion behavior under conditions closer to the actual application environment. Therefore, the aggregation observed in SEM images is likely due to sample-preparation artifacts rather than intrinsic instability of the nanoformulation.

This behavior has been previously reported for chitosan-based delivery systems, where the incorporation of hydrophobic compounds influences particle organization and surface characteristics, resulting in improved morphological definition. Similar morphological features have been reported for EO-loaded NC intended for biomedical applications, including anticancer therapies, by Rajivgandhi *et al*. [26]. Additionally, Taskin *et al*. [27] demonstrated that chitosan nanostructures are suitable carriers for EOs, highlighting their potential across a wide range of biomedical and biotechnological applications.

### *In vitro* cell line response and cytotoxicity

The MTT assay allowed the establishment of a therapeutic margin at 50 μL per well for all evaluated formulations. The MTT assay is widely used to assess the cytotoxic effects of EO-based systems, including oils derived from thyme and sage, due to its sensitivity in detecting alterations in mitochondrial metabolic activity [28, 29].

Analysis of the actin cytoskeleton revealed well-defined stress fibers in control cultures, characterized by radially oriented filaments extending from the perinuclear region toward the cell cortex. This organization is consistent with the cytoskeletal architecture described by Jardon *et al*. [30], which is associated with preserved cell shape regulation, intercellular interactions, and the formation of focal adhesions [30]. The presence of centrally positioned nuclei and clearly defined cellular contours further indicates the maintenance of cytoarchitectural integrity under control conditions.

In cultures treated with NE, actin organization remained largely comparable to that of control cells, suggesting minimal disruption of cytoskeletal structure. In contrast, exposure to NC and free EOs was associated with the onset of partial depolymerization of actin filaments, particularly within stress fibers. These observations are consistent with the cytotoxicity data obtained by the MTT assay, which showed reduced cell viability for NC and free oils after 48 h, whereas NE maintained cell viability above 70%.

Previous studies have reported that reducing EO droplet size can enhance antimicrobial activity by increasing surface area and facilitating interaction with microbial membranes [31]. At the same time, nanoformulation may allow controlled release of bioactive compounds, thereby reducing direct cellular exposure and minimizing cytotoxic effects. Consistent with this concept, the present study demonstrated that free TTO exhibited higher cytotoxicity upon prolonged exposure, whereas its formulation as NE significantly reduced cytotoxicity and preserved cellular metabolic activity.

Although MARC-145 cells were used as a preliminary epithelial model for cytotoxicity screening, this choice was based on their well-established use in evaluating general cytocompatibility, reproducibility, and robustness in *in vitro* assays. These cells provide a reliable first approach for assessing the potential cytotoxic effects of novel formulations before progressing to more specialized models.

Taken together, these findings suggest that NE-based delivery of TTO represents a biocompatible approach that balances antimicrobial efficacy with reduced cytotoxicity, supporting its potential application as a natural therapeutic alternative for the management of bacterial diseases in animals.

### Franz cell release under bovine mammary gland pH conditions

The release profiles revealed a distinct pH-dependent behavior for NC loaded with TTO and thymol. During the initial burst phase, NC exposed to pH 6.6 exhibited a markedly lower release (~5% within the first 5 h) compared with those dispersed at pH 7.2, which showed a higher initial release (>35%). This burst effect is commonly attributed to the rapid diffusion of EO molecules weakly absorbed onto the surface of the polymeric matrix [31].

Release kinetics were further analyzed using the Korsmeyer–Peppas model, which relates the logarithm of cumulative release to the logarithm of time and allows classification of transport mechanisms based on the release exponent (n). For NC, n values indicated non-Fickian (anomalous) transport, reflecting the combined contribution of diffusion and polymer chain relaxation. In contrast, NE exhibited n values ≤0.43, consistent with Fickian diffusion, where release is primarily driven by concentration gradients.

The kinetic constant (k), which reflects the release rate, differed between formulations and pH conditions. For NE, higher k values were observed at pH 6.6, indicating faster diffusion under mildly acidic conditions. Conversely, NC exhibited lower k values at pH 6.6 and faster release at pH 7.2, suggesting reduced matrix cohesion and enhanced diffusion under mastitis-associated pH conditions. Importantly, the release of the active compounds did not require complete degradation of the polymeric matrix.

These findings are consistent with previous reports on EO-loaded chitosan systems prepared by ionic gelation. Shetta *et al*. [32] described similar sustained-release behavior for mint and green tea EOs encapsulated in chitosan matrixes [32]. The slower release phase observed for NC can be attributed to diffusion of encapsulated EO molecules from the polymeric core through interconnected pores and channels, combined with the hydrophobic nature of EOs, which limits water penetration and slows polymer relaxation.

From a therapeutic perspective, the accelerated release of NC at pH 7.2, which simulates intramammary infection, is a potentially advantageous feature [33, 34]. During mastitis, biochemical alterations in the mammary gland, including changes in ionic composition and pH, can compromise tissue integrity and promote bacterial proliferation. A formulation capable of releasing higher amounts of active compounds under these conditions may enhance early antimicrobial availability at the infection site, supporting rapid bacterial containment and reducing disease progression.

In contrast, the more sustained release at physiological pH (6.6) may help maintain local stability and minimize unnecessary exposure of healthy tissue to high concentrations of bioactive agents. Another relevant aspect of the present study is the pH-responsive release behavior observed for NC. During bovine mastitis, milk pH typically increases from approximately 6.5–6.7 to values approaching 7.0–7.4 due to inflammatory processes and leakage of serum components into the mammary gland. The accelerated release observed at pH 7.2 suggests that NC may preferentially release higher concentrations of antimicrobial compounds under infection-associated conditions. This pH-dependent behavior could represent a targeted therapeutic advantage, allowing the system to deliver a rapid antimicrobial burst during infection while maintaining a slower release under physiological conditions.

### Antibacterial activity against mastitis pathogens (*E. coli* and *S. aureus*)

The antibacterial assays revealed a clear formulation-dependent hierarchy of efficacy, with NC showing the strongest inhibitory effect, followed by NP, NE, and free EOs. This trend was consistently observed for *E. coli* and *S. aureus*, although the magnitude of inhibition differed between bacterial species.

The intrinsic antibacterial activity of TTO and thymol against mastitis-associated pathogens has been previously demonstrated by our research group using a broader panel of field-isolated strains [9]. In that study, free EOs showed relevant antibacterial activity; however, variability among strains and limitations related to volatility, stability, and sustained efficacy were also identified.

Encapsulation within NC significantly improved antibacterial performance compared with free oils and NE, supporting the role of the polymeric matrix in protecting, stabilizing, and retaining the active compounds at the bacterial interface. Similar behavior has been reported for chitosan-based delivery systems, in which encapsulation promotes sustained antimicrobial activity and enhanced interaction with bacterial strains [25].

Against *S. aureus*, 0.3% NC exhibited an inhibitory effect comparable to erythromycin. For *E. coli*, erythromycin remained the most effective treatment, although NC showed the highest activity among non-antibiotic formulations. A stronger inhibitory effect was consistently observed against *S. aureus* compared with *E. coli*, which can be attributed to structural differences in bacterial cell envelopes [35].

Both TTO and thymol exert antibacterial effects through membrane destabilization, ion leakage, and interference with respiratory processes, while chitosan contributes through electrostatic interactions with negatively charged bacterial surfaces [35, 36]. The synergistic contribution of chitosan and encapsulated EOs likely underlies the superior efficacy observed for NC [37].

Taken together, these findings demonstrate that nanoencapsulation does not replace the intrinsic antibacterial activity of TTO and thymol, but rather potentiates, stabilizes, and prolongs their effect. The combined encapsulation of TTO and thymol may also enhance antimicrobial performance through their complementary mechanisms of action. Although formal synergy testing, such as fractional inhibitory concentration indices, was beyond the scope of the present study, the enhanced antibacterial activity observed for NC supports the hypothesis that co-delivery of these compounds may potentiate their antimicrobial action.

## CONCLUSION

This study demonstrated that chitosan-based NC loaded with TTO and thymol can enhance the antimicrobial performance of EOs while supporting controlled, pH-responsive release under mastitis-simulated conditions. The NC exhibited suitable physicochemical properties, including submicron PS, low PDI, a positive ZP, and high EE, indicating a stable and reproducible nanoformulation. The release profile was strongly influenced by pH, with limited release at pH 6.6 and increased release at pH 7.2, suggesting preferential availability of active compounds under infection-associated mammary gland conditions. In antibacterial assays, NC produced the greatest inhibitory effect among non-antibiotic formulations, reducing the growth of *S. aureus* and *E. coli* by 92% and 72%, respectively. The activity against *S. aureus* was comparable to erythromycin under the tested conditions.

These findings have practical implications for bovine mastitis management, as pH-responsive NC may improve the local delivery of natural antimicrobial compounds during intramammary infection while reducing unnecessary exposure under physiological conditions. Such a strategy may support antimicrobial stewardship by providing a complementary non-antibiotic approach that could reduce dependence on conventional antibiotics in dairy production systems.

The main strength of this study is the integrated evaluation of formulation properties, cytocompatibility, release kinetics, and antibacterial activity using mastitis-relevant pH conditions and representative bacterial pathogens. However, the study was limited to *in vitro* assays, reference bacterial strains, and controlled laboratory conditions. Milk matrix stability, interaction with mammary tissue, pharmacokinetics, safety, and therapeutic efficacy were not evaluated.

Future studies should validate these NC in milk matrixes, mammary epithelial models, *ex vivo* udder tissue systems, and *in vivo* mastitis models. Further work should also include MIC, MBC, viable bacterial counts, synergy testing, long-term stability, dose optimization, and safety assessment before clinical application.

Overall, chitosan-based NC loaded with TTO and thymol represent a promising pH-responsive delivery platform for mastitis-associated pathogens and warrant further development as part of integrated strategies to reduce antibiotic use in dairy cattle.

## DATA AVAILABILITY

The data generated during the study are included in the manuscript.

## AUTHORS’ CONTRIBUTIONS

LCG conceived and designed the study. LCG performed the formulation of nanocapsules, physicochemical characterization, and in vitro assays. ADR conducted the scanning electron microscopy analysis. SJX performed the cytotoxicity assays. LHA conducted the bacteriological assays. LMA conducted the statistical data analysis. MLZZ contributed to the experimental design and data interpretation. DQG supervised, conceived, and funded the study. LCG wrote the manuscript. All authors reviewed and approved the final manuscript.
